# Supplementary material for: Genome-Wide Identification of the Highly Conserved INDETERMINATE DOMAIN (IDD) Zinc Finger Gene Family in Moso Bamboo (Phyllostachys edulis)
Source: Int J Mol Sci. 2022 Nov 12;23(22):13952. doi: 10.3390/ijms232213952 (PMC9695771; doi:10.3390/ijms232213952)
Supplement: Supplementary file 1 [file ijms-23-13952-s001.zip › Table S1.pdf]

Table S1. The information of 32 *PheIDD* genes in moso bamboo

Gene	Gene ID	Identity with ZmID1(%)	Peptide length	Molecular weight(kDa)	PI	Instability index	Aliphatic index	GRAVY	Exons
PheIDD1	PH02Gene23030.t1	64.57	390	42.57	8.67	52.17	73.33	-0.437	4
PheIDD2	PH02Gene16952.t1	49.43	300	33.46	8.94	48.68	71.6	-0.595	6
PheIDD3	PH01000878G0370	39.42	387	41.64	9.27	62.67	61.89	-0.557	3
PheIDD4	PH02Gene13636.t1	36.75	382	41.92	8.73	59.91	61.34	-0.659	3
PheIDD5	PH02Gene39676.t1	35.31	489	51.88	8.91	54.77	58.1	-0.508	3
PheIDD6	PH02Gene22178.t1	34.34	494	52.59	8.72	54.98	56.11	-0.542	3
PheIDD7	PH02Gene42416.t1	34.14	477	50.43	9.15	48.04	71.55	-0.31	3
PheIDD8	PH02Gene14249.t1	34.05	496	53.07	7.05	53.8	60.26	-0.447	3
PheIDD9	PH02Gene14926.t1	33.78	479	50.88	9.02	41.85	63.51	-0.462	3
PheIDD10	PH02Gene32318.t1	33.72	251	27.52	8.96	64.13	66.89	-0.58	2
PheIDD11	PH02Gene01591.t1	33.16	530	55.32	9.02	69.62	61.66	-0.458	4
PheIDD12	PH02Gene39072.t1	32.72	533	55.56	9.49	73.55	57.13	-0.49	4
PheIDD13	PH02Gene09285.t1	32.58	522	54.61	9.42	48.61	52.49	-0.532	3
PheIDD14	PH02Gene28491.t1	32.34	237	26.13	8.27	53.93	63.84	-0.706	2

Table S1. *Cont.*

Gene	Gene ID	Identity with ZmID1(%)	Peptide length	Molecular weight(kDa)	PI	Instability index	Aliphatic index	GRAVY	Exons
PheIDD15	PH02Gene47146.t1	32.31	542	56.5	9.4	46.69	53.99	-0.508	3
PheIDD16	PH02Gene06891.t1	32.16	480	50.66	8.97	51.41	69.71	-0.313	3
PheIDD17	PH02Gene48728.t1	31.2	548	57.39	9.17	49.06	53.36	-0.49	3
PheIDD18	PH02Gene30315.t1	31.07	553	57.86	9.48	46.96	57.11	-0.461	3
PheIDD19	PH02Gene01518.t2	30.73	587	61.13	9.15	43.75	60.02	-0.418	3
PheIDD20	PH02Gene23446.t1	30.18	414	43.74	9.44	68.64	68.64	-0.479	3
PheIDD21	PH02Gene01883.t2	30.03	586	60.97	8.87	43.55	58.81	-0.422	3
PheIDD22	PH02Gene06314.t1	29.68	442	46.65	8.78	42.59	64.21	-0.407	4
PheIDD23	PH02Gene19118.t1	29.48	408	43.07	9.36	68.68	65	-0.498	3
PheIDD24	PH02Gene48787.t1	29.36	490	53.96	7.05	62.39	59.98	-0.683	3
PheIDD25	PH02Gene00552.t1	28.04	591	61.33	8.77	43.08	60.29	-0.408	3
PheIDD26	PH02Gene13399.t1	28.01	579	60.58	9.14	43.53	59.14	-0.437	3
PheIDD27	PH02Gene39810.t1	27.24	493	53.02	8.77	66.29	65.52	-0.505	3
PheIDD28	PH02Gene38704.t1	26.48	486	52.29	9.07	58.04	66.46	-0.528	3

Table S1. *Cont.*

Gene	Gene ID	Identity with ZmID1(%)	Peptide length	Molecular weight(kDa)	PI	Instability index	Aliphatic index	GRAVY	Exons
PheIDD29	PH02Gene22093.t3	26.1	490	52.64	8.92	60.01	64.12	-0.473	3
PheIDD30	PH02Gene37005.t1	26.06	494	53.17	9.18	62.1	66.94	-0.492	3
PheIDD31	PH02Gene13322.t1	26.02	621	66.67	6.11	62.81	63.85	-0.539	6
PheIDD32	PH02Gene17906.t1	25.56	595	63.33	5.33	60.59	63.55	-0.531	4
