# Supplementary material for: Genome-Wide Identification of the Highly Conserved INDETERMINATE DOMAIN (IDD) Zinc Finger Gene Family in Moso Bamboo (Phyllostachys edulis)
Source: Int J Mol Sci. 2022 Nov 12;23(22):13952. doi: 10.3390/ijms232213952 (PMC9695771; doi:10.3390/ijms232213952)
Supplement: Supplementary file 1 [file ijms-23-13952-s001.zip › Table S2.pdf]

Table S2 Genes co-expressed with PheIDD21

co-expressed gene	wPCC	MR (Mutual rank)
PH02Gene04877	0.99	1.00
PH02Gene11386	0.98	2.00
PH02Gene17121	0.96	1.00
PH02Gene35441	0.96	2.00
PH02Gene50527	0.96	3.00
PH02Gene41334	0.96	2.00
PH02Gene19556	0.96	3.87
PH02Gene13505	0.95	4.24
PH02Gene21067	0.95	3.74
PH02Gene33706	0.95	6.32
