# Supplementary material for: Genome-Wide Identification of the Highly Conserved INDETERMINATE DOMAIN (IDD) Zinc Finger Gene Family in Moso Bamboo (Phyllostachys edulis)
Source: Int J Mol Sci. 2022 Nov 12;23(22):13952. doi: 10.3390/ijms232213952 (PMC9695771; doi:10.3390/ijms232213952)
Supplement: Supplementary file 1 [file ijms-23-13952-s001.zip › Table S3.pdf]

Table S3. Primers used in this study

Name	Purpose	Forward primer Sequence(5'to3')	Reverse primer Sequence(5'to3')
PheIDD1	qRT-PCR	CTTCCTCCCTTTCTCCAACC	CGTAAGTAAGCCCGAAGCTG
PheIDD2	qRT-PCR	TGACGGGGATAAAGAAGCAC	TCAGCTAGGGCATCACAGAA
PheIDD3	qRT-PCR	ACAACCTGCCGTGGAAGCTCAA	CGTGCTTGCGACTGAAGTGCTT
PheIDD4	qRT-PCR	ACCTACCCCAACCATCTCAGTGC	ACGTGTACCTCTCAAAGATAACCC
PheIDD5	qRT-PCR	AAGAAGTACGCCGTCCACTCCG	TGGTGGTCGTAGGGCATAGTGG
PheIDD6	qRT-PCR	ACAACCTGCCGTGGAAGCTGAA	CTGGAGAAGATGGTGCCGCAGT
PheIDD7	qRT-PCR	GTGCAGCAAGTTCGACAGTGGC	TTGCACGCCAACACCCAAGAAA
PheIDD8	qRT-PCR	AGACAGCTTCGTGACTCACCGC	TCTTGATGTCCGGGGCGAATCC
PheIDD9	qRT-PCR	CGCGACCATCAATGCAGCAACA	GACGTTGACAGGCAGGGATCGA
PheIDD10	qRT-PCR	TCTTCACCAGGAGGGACAGCTT	GTTGTCTGGCAGCATCGACAGT
PheIDD11	qRT-PCR	CAGAGGGTTCACTGCCGCAATG	TTGGATGAATGCGGAGAGGCCC
PheIDD12	qRT-PCR	AGCCATGCAAGTGTCAATCC	GGCCCAGAAAGTCCAATGTG
PheIDD13	qRT-PCR	AGGCGTCCTCCTCCTCGTTCTA	TTGTACAGGGAAGGGAAGCCGG
PheIDD14	qRT-PCR	GAGCCGGCCAAGAAGAAGAGGA	AACCCCTTACCACACACCTCGC
PheIDD15	qRT-PCR	GACCTCATCGTCGTCCTCTC	TGAGGAGGCTGTTATTCCCC
PheIDD16	qRT-PCR	GAGTGCAGCAAGTTCGACGGTG	GCCGACGCCCAAGAAATCCCTA
PheIDD17	qRT-PCR	GGCGAGAAGAAGTGGAAGTGCG	TTGGAAGGTCGGCAGTTGGGAG
PheIDD18	qRT-PCR	CCCTTCCCTGTACAACTCGT	GCCAGCGAGTTCATGATGAG
PheIDD19	qRT-PCR	AGGTTCTGAGACATCGGCTGCG	GGCCTGCTCACTGTACATCCCA
PheIDD20	qRT-PCR	AACACTTAGCAGGGGGTGGT	CCCAAAGGAGCAACTAGCAC
PheIDD21	qRT-PCR	GCGGAAACTTTGTTGGTGGCGA	CCAGCGAGCGAGTTCATCAGGT
PheIDD22	qRT-PCR	GCCTTCTCCCACAGCGACATA	TTGCTGCTCTGGTGTCCTTGCT
PheIDD23	qRT-PCR	ATGCACCTTCACCTTTCCAC	ATTTGGTGGTGGCTCTTGAC
PheIDD24	qRT-PCR	CTGAGATCTTTATCCCCATATGCT	TTGGGAAGCCTTTTAGAAGTAATG
PheIDD25	qRT-PCR	GTTGGTGGAGATCATGTCGCGC	CCAGCGAGCGAGTTCATCAGGT
PheIDD26	qRT-PCR	ACGGAGGGACAGCTTCATCACC	AACCCCGATGCTTGTGGAGGAG
PheIDD27	qRT-PCR	CTGCTCCAGATCACCTGCTT	TGATGTCCATCCTAGCGTGT
PheIDD28	qRT-PCR	CAACGAGGCTGAAGGAGGATG	GTGTTGAGCTGATGGTGGTCGT
PheIDD29	qRT-PCR	GATCACCTGTTTCAGCTGCC	TGTCCATCCTAGCGTGTTGG
PheIDD30	qRT-PCR	GTTCGCGGTGGAGTAGGAG	CGTGGAATGCTGCGATGTTA
PheIDD31	qRT-PCR	TCCCCTCACCCTCTGAAGCCAT	CGCCGATAAGATCACGCAGGGA
PheIDD32	qRT-PCR	TTCGTCCTCCTCATCAACCC	TTCCCACTGTCATAGGCGAG
PheNTB	Internal standard	TCTTGTTTGACACCGAAGAGGAG	AATAGCTGTCCCTGGAGGAGTTT
PheIDD3	gene clone	TCTAAACACTTACTGCAATTGCTG	TTCCACATCGAACTAGCTTACCT
PheIDD5	gene clone	CAGCATGTCCAACCTCACCT	TGCGAGGAAGAGGAAAGC

Table S3. *Cont.*

PheIDD6	gene clone	GATGGATGGATGCGAAATACTC	TGATCAGATGCAAGGTTGATG
PheIDD11	gene clone	TGCGTGGGTGTTGGTTTG	GGGCGTAACCTGGCTTCTACTA
PheIDD13	gene clone	AGATGTCTATGGCGTATCTATCTCG	CTTCTTGATCTCTTGCTGTGATG
PheIDD17	gene clone	AGAGAACAACACAAGGTAGCGG	AAGGACGAGAAGATGGTGCC
PheIDD25	gene clone	GTGTTTGAGGTGGTGTGAGC	TTGAAGGACGAAGCCGACT
PheIDD26	gene clone	GGTTTCCCTGTTGTTGTTGA	ATCCTGCCTCCATTATTGAAG
PheIDD27	gene clone	CAAGATAAAGAAACAAGCGTGC	TACACCACACCTCACCCTG
PheIDD28	gene clone	GCAGTGCCACTATGTTGAGTTC	CACATCCCAGCCCATTGAT
PheIDD31	gene clone	ATCTCCGTCCACCACTCG	GAAGCATCGATCTATATACAGGTC
BD-PheIDD21 α/β	Yeast two-hybrid	ggccgaattcccggggATGGCATCGAATTCATCGGCTG	ccgctgcaggtcgacggatccTCATGGCATCCTGCCGCCG
AD-PheIDD21 α/β	Yeast two-hybrid	gagtggccattatggcccgggATGGCATCGAATTCATCGGCTG	gccgacatgtttttcccgggTCATGGCATCCTGCCGCCG
AD-PheDELLAa	Yeast two-hybrid	gagtggccattatggcccgggATGAAGCGCGAGTACCAAGACGC	gccgacatgtttttcccgggTCACGCTGCGGCGACGCGCCAT
AD-PheDELLAb	Yeast two-hybrid	gagtggccattatggcccgggATGAAGCGCGAGTACCAAGACGC	gccgacatgtttttcccgggTCACGCTGCGGCGTCGCGCCA
AD-PheSCL3a	Yeast two-hybrid	gagtggccattatggcccgggATGGTACATGACGAAGGCTCC	gccgacatgtttttcccgggTCAGTCGAACCTGCGGCC
pHis-17121A	yeast one-hybrid	attcccggggagctcacgcgtGTCGGATGAAGTGCGCCATG	cggatcgattcgcgaacgcgtCATCTCTCTCACACCCGCACA
pHis-17121B	yeast one-hybrid	attcccggggagctcacgcgtTCGTCTTCCTCTTTACCACG	cggatcgattcgcgaacgcgtTTTGGTCACGCCATAACCAA
pHis-17121C	yeast one-hybrid	attcccggggagctcacgcgtAGTGAGGCCATTTCCCATGC	cggatcgattcgcgaacgcgtTCGCACTTATGGCTACCACT
pHis-17121D	yeast one-hybrid	attcccggggagctcacgcgtTATGAGGTGCCAAGACGAGG	cggatcgattcgcgaacgcgtCAGGCCAAAGAGAAGAGAGGC
pHis-35441A	yeast one-hybrid	attcccggggagctcacgcgtTGTGCCGTGAACATTCTGGT	cggatcgattcgcgaacgcgtAGCAGTTGACTCCGCTTACT
pHis-35441B	yeast one-hybrid	attcccggggagctcacgcgtAGCCGCTGATCGGAAATTGC	cggatcgattcgcgaacgcgtTGGTGCTCCATATTGAGACT
pHis-35441C	yeast one-hybrid	attcccggggagctcacgcgtAAACCCAGTAGGAGGAAGGT	cggatcgattcgcgaacgcgtCTTGGGTTGCGATCTGGCAT
pHis-11386A	yeast one-hybrid	attcccggggagctcacgcgtTCAAGTGCAATCTAATCACG	cggatcgattcgcgaacgcgtCTAGCGGTTGCACAGATGCG
pHis-11386B	yeast one-hybrid	attcccggggagctcacgcgtTGGCGGGTAAACATGCGTTG	cggatcgattcgcgaacgcgtGGTTGCCTGGTCACTCTCTT
