# Supplementary material for: Genome-Wide Identification of the Highly Conserved INDETERMINATE DOMAIN (IDD) Zinc Finger Gene Family in Moso Bamboo (Phyllostachys edulis)
Source: Int J Mol Sci. 2022 Nov 12;23(22):13952. doi: 10.3390/ijms232213952 (PMC9695771; doi:10.3390/ijms232213952)
Supplement: Supplementary file 1 [file ijms-23-13952-s001.zip › Table S4.pdf]

Table S4. List of IDD genes identified in the sequence databases

Species	Accession number	Gene	Datebase
Arabidopsis thaliana	AT5G66730.1	AtIDD1	Phytozome
	AT3G50700.1	AtIDD2	Phytozome
	AT1G03840.1	AtIDD3	Phytozome
	AT2G02080.1	AtIDD4	Phytozome
	AT2G02070.1	AtIDD5	Phytozome
	AT1G14580.1	AtIDD6	Phytozome
	AT1G55110.1	AtIDD7	Phytozome
	AT5G44160.2	AtIDD8	Phytozome
	AT3G45260.1	AtIDD9	Phytozome
	AT5G03150.1	AtIDD10	Phytozome
	AT3G13810.1	AtIDD11	Phytozome
	AT4G02670.1	AtIDD12	Phytozome
	AT5G60470.1	AtIDD13	Phytozome
	AT1G68130.1	AtIDD14	Phytozome
	AT2G01940.1	AtIDD15	Phytozome
	AT1G25250.1	AtIDD16	Phytozome
Zea mays	GRMZM2G011357	ZmID1	Phytozome
	GRMZM2G465595	ZmIDD18	Phytozome
	GRMZM2G074032	ZmIDD16	Phytozome
	GRMZM2G123094	ZmIDD15	Phytozome
	GRMZM2G141031	ZmIDD14	Phytozome
	GRMZM2G110107	ZmIDD17	Phytozome
	GRMZM2G171073	ZmIDD1	Phytozome
	GRMZM2G058197	ZmIDD10	Phytozome
	GRMZM2G022213	ZmIDD8	Phytozome
	GRMZM2G046290	ZmIDD5	Phytozome
	GRMZM2G143723	ZmIDD2	Phytozome
	GRMZM2G177693	ZmIDD11	Phytozome
	GRMZM2G027333	ZmIDD12	Phytozome
	GRMZM2G320287	ZmIDD7	Phytozome
	GRMZM2G021587	ZmIDD13	Phytozome
	GRMZM2G042666	ZmIDDveg7	Phytozome
	GRMZM2G179677	ZmIDDp1	Phytozome
	GRMZM2G090595	ZmIDDp10	Phytozome

Table S4. *Cont.*

	GRMZM5G884137	ZmIDD9	Phytozome
	GRMZM2G129261	ZmIDDveg9	Phytozome
	GRMZM5G828179	ZmIDD3	Phytozome
	GRMZM2G151309	ZmIDD4	Phytozome
	GRMZM2G035625	ZmIDD6	Phytozome
	Os10g28330	OsID1	Phytozome
	Os03g10140	OsIDD1	Phytozome
	Os01g09850	OsIDD2	Phytozome
	Os09g38340	OsIDD3	Phytozome
	Os02g45054	OsIDD4	Phytozome
	Os07g39310	OsIDD5	Phytozome
Oryza sativa	Os08g44050	OsIDD6	Phytozome
	Os02g31890	OsIDD7	Phytozome
	Os01g14010	OsIDD8	Phytozome
	Os01g70870	OsIDD9	Phytozome
	Os04g47860	OsIDD10	Phytozome
	Os01g39110	OsIDD11	Phytozome
	Os08g36390	OsIDD12	Phytozome
	Os09g27650	OsIDD13	Phytozome
	Os03g13400	OsIDD14	Phytozome
	PH02Gene23030.t1	PheIDD1	Zhao et al. 2018
	PH02Gene16952.t1	PheIDD2	Zhao et al. 2018
	PH01000878G0370	PheIDD3	Zhao et al. 2018
	PH02Gene13636.t1	PheIDD4	Zhao et al. 2018
	PH02Gene39676.t1	PheIDD5	Zhao et al. 2018
	PH02Gene22178.t1	PheIDD6	Zhao et al. 2018
	PH02Gene42416.t1	PheIDD7	Zhao et al. 2018
	PH02Gene14249.t1	PheIDD8	Zhao et al. 2018
	PH02Gene14926.t1	PheIDD9	Zhao et al. 2018
	PH02Gene32318.t1	PheIDD10	Zhao et al. 2018
	PH02Gene01591.t1	PheIDD11	Zhao et al. 2018
Phyllostachys edulis	PH02Gene39072.t1	PheIDD12	Zhao et al. 2018
	PH02Gene09285.t1	PheIDD13	Zhao et al. 2018
	PH02Gene28491.t1	PheIDD14	Zhao et al. 2018
	PH02Gene47146.t1	PheIDD15	Zhao et al. 2018
	PH02Gene06891.t1	PheIDD16	Zhao et al. 2018
	PH02Gene48728.t1	PheIDD17	Zhao et al. 2018

Table S4. *Cont.*

	PH02Gene30315.t1	PheIDD18	Zhao et al. 2018
	PH02Gene01518.t2	PheIDD19	Zhao et al. 2018
	PH02Gene23446.t1	PheIDD20	Zhao et al. 2018
	PH02Gene01883.t2	PheIDD21	Zhao et al. 2018
	PH02Gene06314.t1	PheIDD22	Zhao et al. 2018
	PH02Gene19118.t1	PheIDD23	Zhao et al. 2018
	PH02Gene48787.t1	PheIDD24	Zhao et al. 2018
	PH02Gene00552.t1	PheIDD25	Zhao et al. 2018
	PH02Gene13399.t1	PheIDD26	Zhao et al. 2018
	PH02Gene39810.t1	PheIDD27	Zhao et al. 2018
	PH02Gene38704.t1	PheIDD28	Zhao et al. 2018
	PH02Gene22093.t3	PheIDD29	Zhao et al. 2018
	PH02Gene37005.t1	PheIDD30	Zhao et al. 2018
	PH02Gene13322.t1	PheIDD31	Zhao et al. 2018
	PH02Gene17906.t1	PheIDD32	Zhao et al. 2018
