# Supplementary figures and images for: Genome-Wide Identification of the Highly Conserved INDETERMINATE DOMAIN (IDD) Zinc Finger Gene Family in Moso Bamboo (Phyllostachys edulis)
Source: Int J Mol Sci. 2022 Nov 12;23(22):13952. doi: 10.3390/ijms232213952 (PMC9695771; doi:10.3390/ijms232213952)

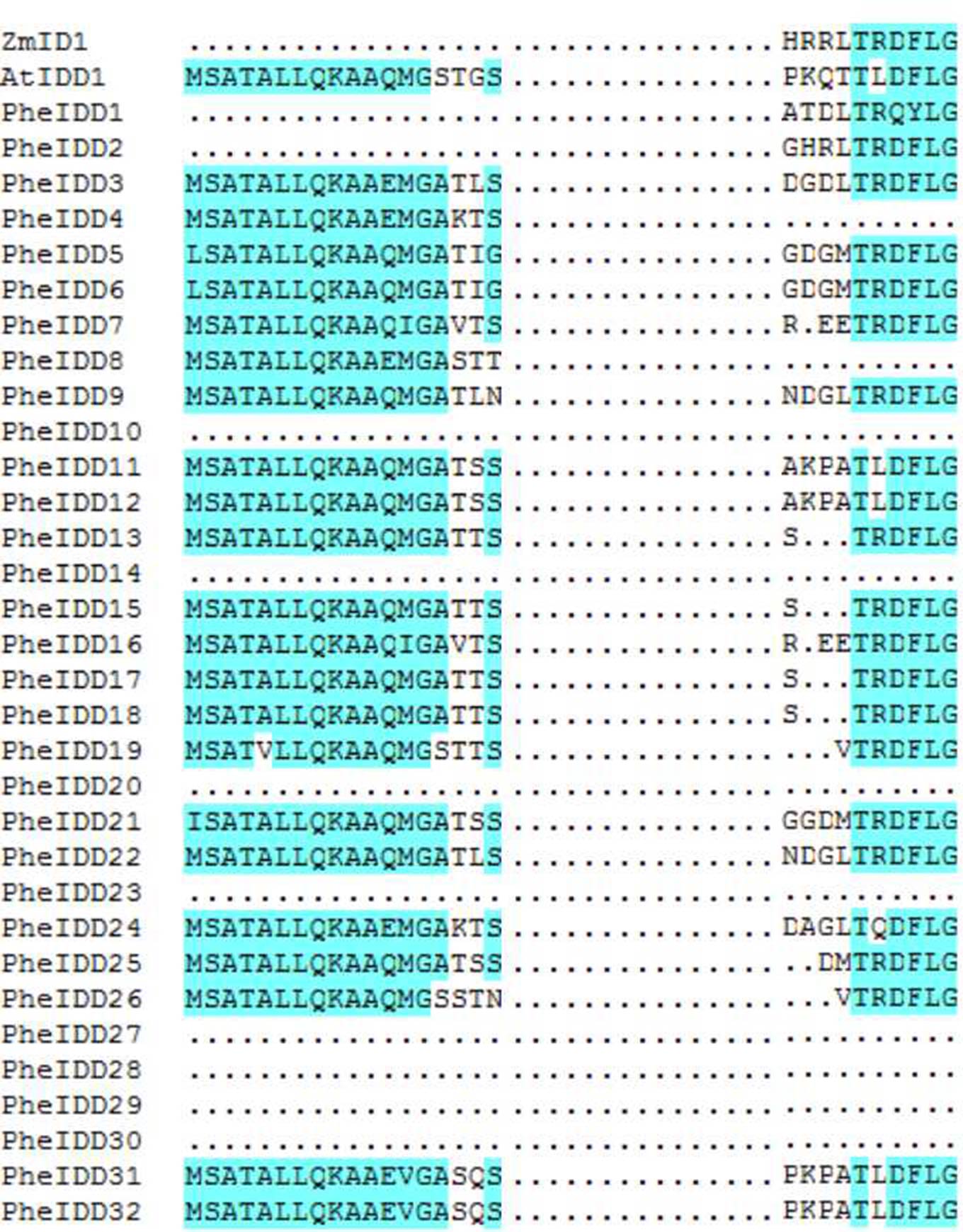

Supplement: Supplementary file 1 [file ijms-23-13952-s001.zip › Figure S2.jpg]

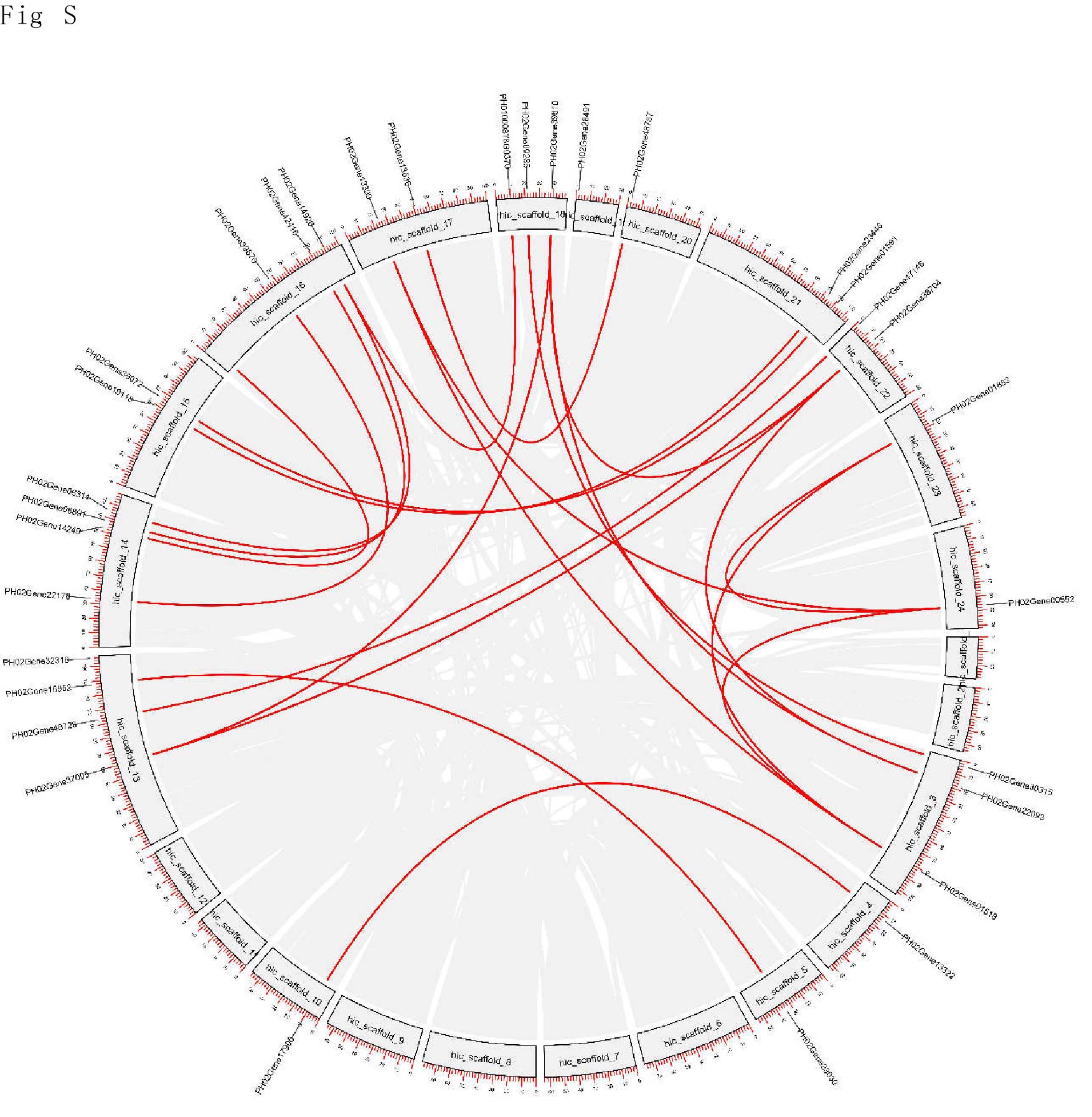

Supplement: Supplementary file 1 [file ijms-23-13952-s001.zip › Figure S4.jpg]

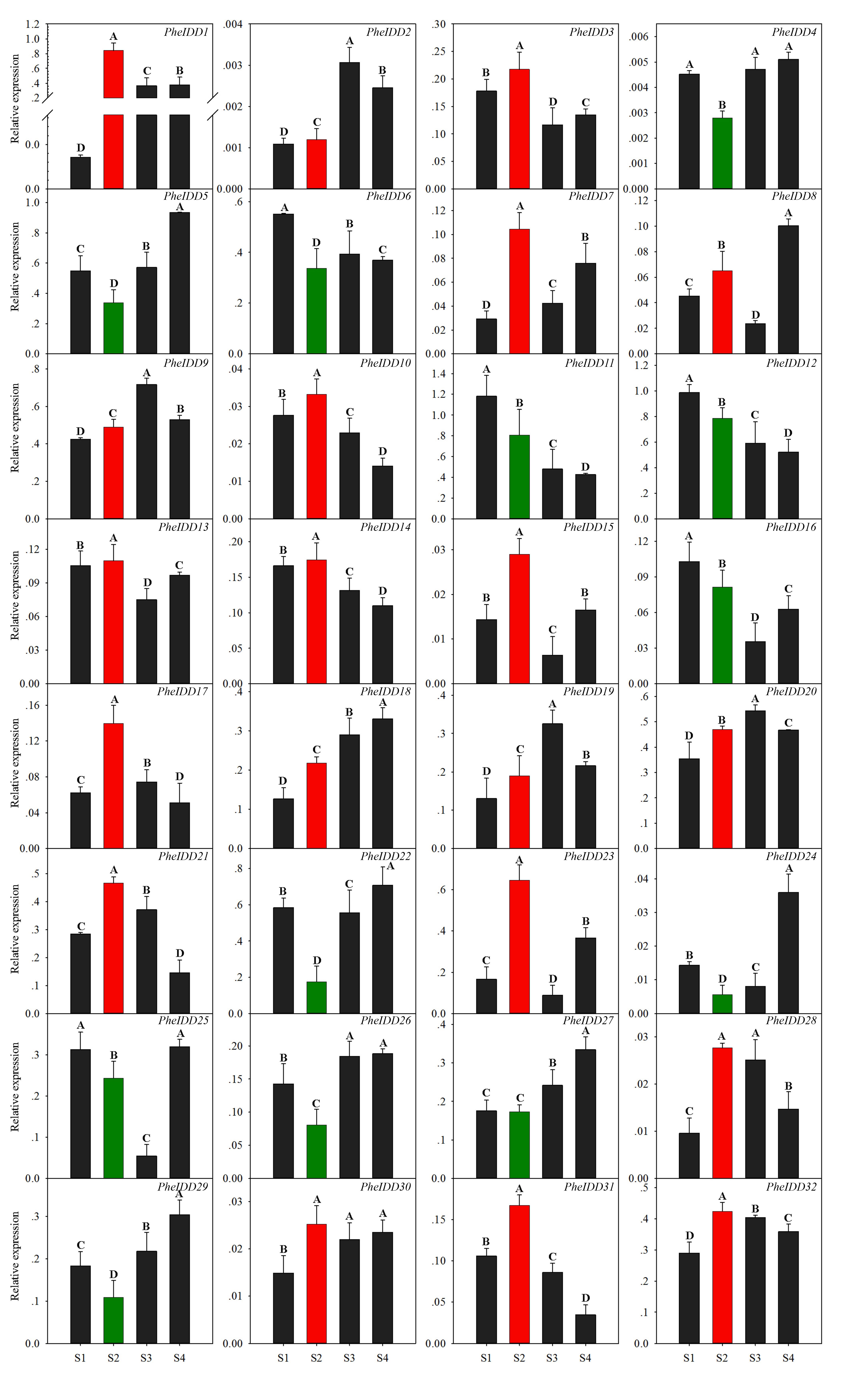

Supplement: Supplementary file 1 [file ijms-23-13952-s001.zip › Figure S5.jpg]

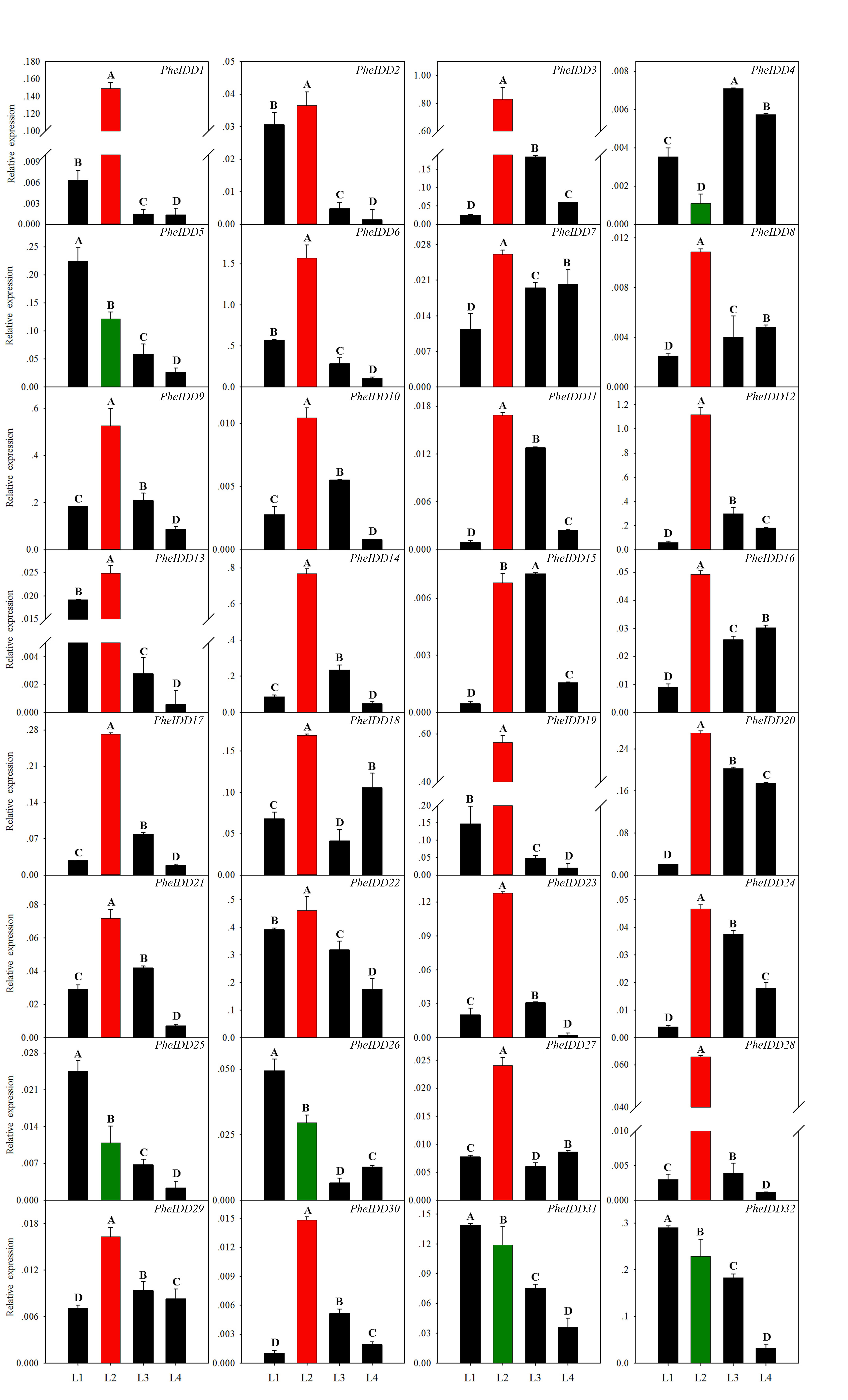

Supplement: Supplementary file 1 [file ijms-23-13952-s001.zip › Figure S6.jpg]

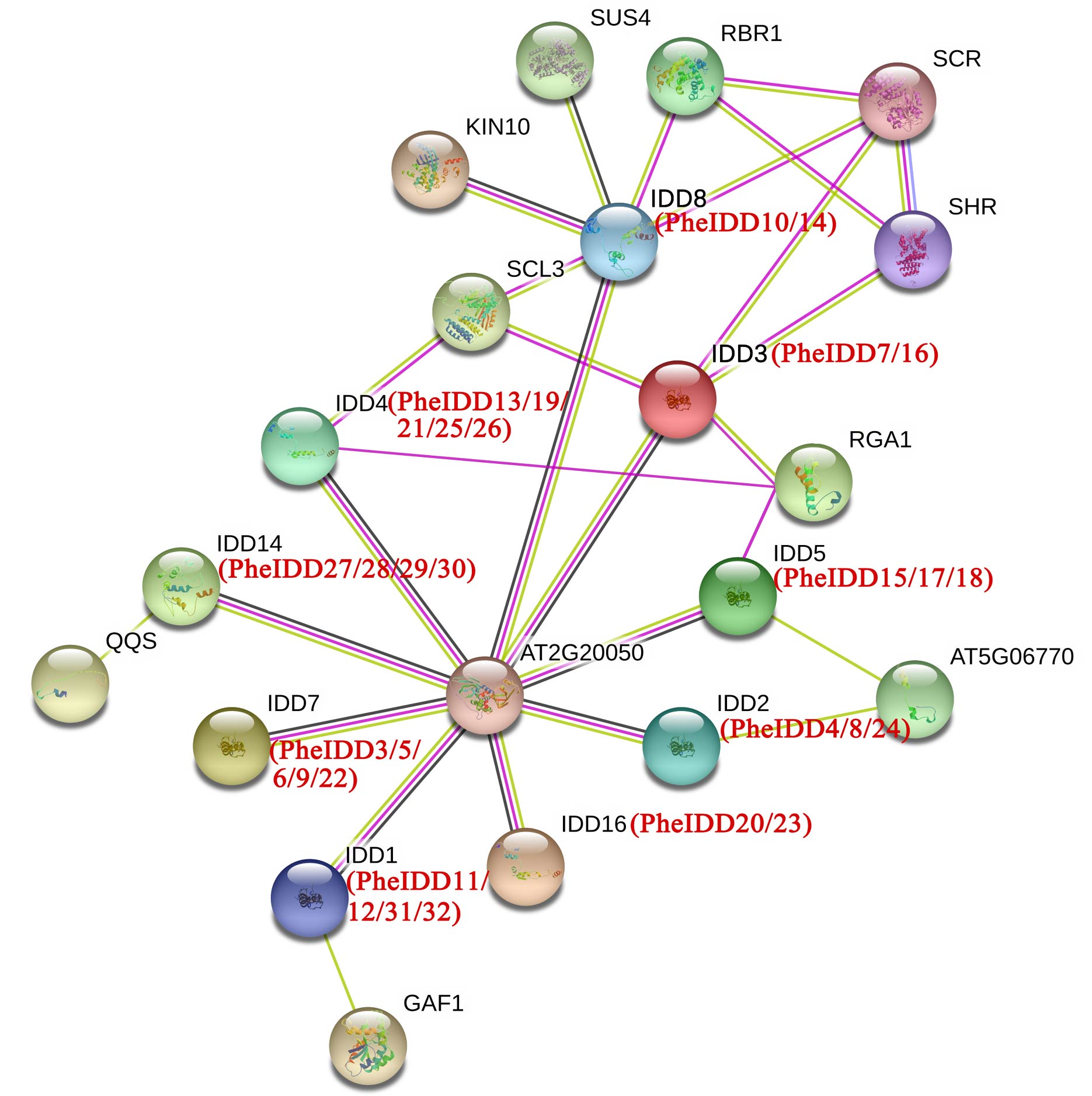

Supplement: Supplementary file 1 [file ijms-23-13952-s001.zip › Figure S7.jpg]
